# Role of Dendritic Cells in Inflammation and Loss of Tolerance in the Elderly

**DOI:** 10.3389/fimmu.2017.00896

**Published:** 2017-07-26

**Authors:** Anshu Agrawal, Sudhanshu Agrawal, Sudhir Gupta

**Affiliations:** ^1^Division of Basic and Clinical Immunology, Department of Medicine, University of California, Irvine, Irvine, CA, United States

**Keywords:** dendritic cells, inflammation, tolerance, aging, mucosa

## Abstract

Dendritic cells (DCs) play an important role in advancing age-associated progressive decline in adaptive immune responses, loss of tolerance, and development of chronic inflammation. In aged humans, DCs secrete increased levels of pro-inflammatory cytokines and decreased levels of anti-inflammatory and immune-regulatory cytokines. This may contribute to both chronic inflammation and loss of tolerance in aging. Aged DCs also display increased immune response against self-antigens contributing further to both inflammation and loss of tolerance. The secretion of innate protective cytokines such as type I and III interferons is decreased, and the function of DCs in airway remodeling and inflammation in aged is also compromised. Furthermore, the capacity of DCs to prime T cell responses also seems to be affected. Collectively, these changes in DC functions contribute to the immune dysfunction and inflammation in the elderly. This review only focuses on age-associated changes in DC function in humans.

## Introduction

Medical and technological advances have significantly enhanced the life expectancy of the human population (1–3). As a result, age expectancy has increased but is accompanied by a substantial increase in age-related diseases including cardiovascular, neurodegenerative disorders, infections, autoimmunity, diabetes, and cancers. Age-related immune dysfunction contributes to the increased incidence of these diseases due to impaired surveillance, repair, and regulation (4, 5). Advanced age affects the functions of both the innate and the adaptive arms of the immune system. Changes in adaptive immune T and B cells are more apparent as there is decline in cell numbers as well as functions (6). In contrast, functions of innate immune cells, such as dendritic cells (DCs), macrophages do not display major numerical or phenotypic changes; however, significant changes in regulation of the immune responses by these cells exist (7). DCs are well established as the most effective of the antigen-presenting cells (APCs) (8). They express high levels of the molecules that are required for antigen presentation such as the MHC II, CD80, and CD86 on activation (9, 10). DCs are thus highly effective in initiating an immune response (11). DCs are distributed throughout the body, including the mucosal tissues, where they are found below the epithelial cell barrier. DCs present at the mucosal sites, and in tissues, survey for external and internal danger signals using an array of pattern recognition receptors such as the toll-like receptor (TLRs), C-type lectin receptors, NOD-like receptors (NLRs), and others (12). These receptors can sense not only external infectious and environmental antigens but are also capable of responding to internal danger signals and molecules generated during tissue injury or malfunctioning of any of the other processes in the body (13). Following uptake of antigens *via* the PRRs, DCs are activated and migrate to the lymph nodes to present antigens to the T cells, and initiate an adaptive immune response. In contrast, presentation of antigens, particularly self-antigens to T cells by unactivated DC prevents T cell activation and induces tolerance (14). DCs, thus play, a dual role that of generating immunity against danger signals and preventing immunity against self. Since increased susceptibility to infections as well as increased reactivity to self is a characteristic of aging, aberrant DC function can play a major role in age-related disorders. This review therefore focuses on the changes in human DC functions in the aged population.

## DC Numbers and Phenotype

Dendritic cells can be divided into two major subclasses (1) plasmacytoid DCs (pDCs), which are of lymphoid origin and express B plasma cell markers; (2) myeloid DCs (mDCs) that are derived from myeloid progenitors (15). Hematopoiesis in aging is characterized by decrease in lymphoid cells with skewing toward the myeloid lineages (16, 17). In keeping with this, a decrease in pDC numbers in circulation has been observed in the aged population (18–20). Myeloid DC numbers have been reported to be largely unchanged in circulation (18, 20–22). Recent data from DC field have led to the further division of myeloid DC into several subsets (15). However, only two of these are present in the circulation, the CD1c^+^ and the CD141^+^ mDC subsets. We have observed a decrease in the CD141^+^ mDC subset in the circulation of the aged subjects; however, the number of CD1c^+^ mDCs was not affected with age (23). Monocyte-derived DCs (MoDCs) represent a third subset of DCs. MoDCs have similar functions as myeloid DCs in circulation though recent genomic studies suggest that these two populations are significantly different at the transcriptome level (24). The MoDCs numbers are also stable with age (21). The phenotype of the DCs is also reported to be largely unchanged though most of the data are from MoDCs (21, 25–27), and very few studies have examined the phenotype of pDCs and mDCs (18, 20, 22).

Information regarding the age-associated changes in tissue DCs is scarce due to the difficulty in obtaining human samples. Nevertheless, a very recent and comprehensive study by Granot et al. (28) has examined the CD1c^+^ and CD141^+^ mDC distribution, activation, and migration in various organs including the lung and the intestine and associated lymph nodes. The samples were from 78 organ donors of various ethnicities with ages ranging from infants to 93-year-old adults. The authors did not observe significant changes in DC subset frequencies throughout life in most tissues. They did observe a trend toward increased maturation of DCs particularly CD1c^+^ DC subset in the lung, mesenteric, and inguinal lymph nodes indicating increased activation and migration of DCs with age.

## Pathogen Sensing and Response of DCs

A hallmark of advancing age is an increased susceptibility to acute viral and bacterial infections. These infections are often more severe and prolonged, with a higher mortality rate among older adults (29). One of the primary functions of DCs as a part of the innate immune system is to sense and respond to external pathogenic stimuli *via* PRRs such as TLRs. The function of TLRs in aged subjects has been reported to be defective in both mDC and pDC subsets (18).

Among the DC subsets, pDCs play a major role in controlling viral infections *via* secretion of large amounts of type I interferons early in the immune response (30). A decrease in IFN secretion by pDCs from elderly in response to TLR7 and TLR9 ligands as well as to different viruses including influenza has been reported (18, 20, 31, 32). The data on the expression of TLR7 in pDC from aged subjects are rather conflicting; both decreased and normal expression has been reported (18); expression of TLR9 in pDC in elderly is comparable to young subjects. Impaired functions of pDCs are considered a major factor in increased susceptibility of elderly to viral infections.

In addition to pDCs, mDCs also play a major role in defense against microbes. mDCs in circulation express a wide array of PRRs including TLRs. Few studies have examined the expression and functions of TLRs in mDC in humans. Panda et al. (18) have performed an extensive analysis of the intracellular cytokine secretion by mDCs in response to multiple TLR ligands including TLR1/2, TLR2/6, TLR3, TLR4, TLR5, and TLR8. They observed a significant decrease in the production of TNF-α, IL-6, and IL-12p40 against almost all ligands tested. The decrease was consistent and was there even when the subjects were retested after an interval of 2 years. The decrease was attributed in part to reduced expression of TLR1, TLR3, and TLR8. TLR2 and TLR4 expression was reported to be unaltered. Della Bella et al. (33) have also reported a decreased IL-12 production by DCs from peripheral blood mononuclear cells from aged subjects in response to TLR4 ligand as compared to young individuals. There are no studies with purified mDCs probably due to very small number of circulating mDCs.

**Figure 1:**
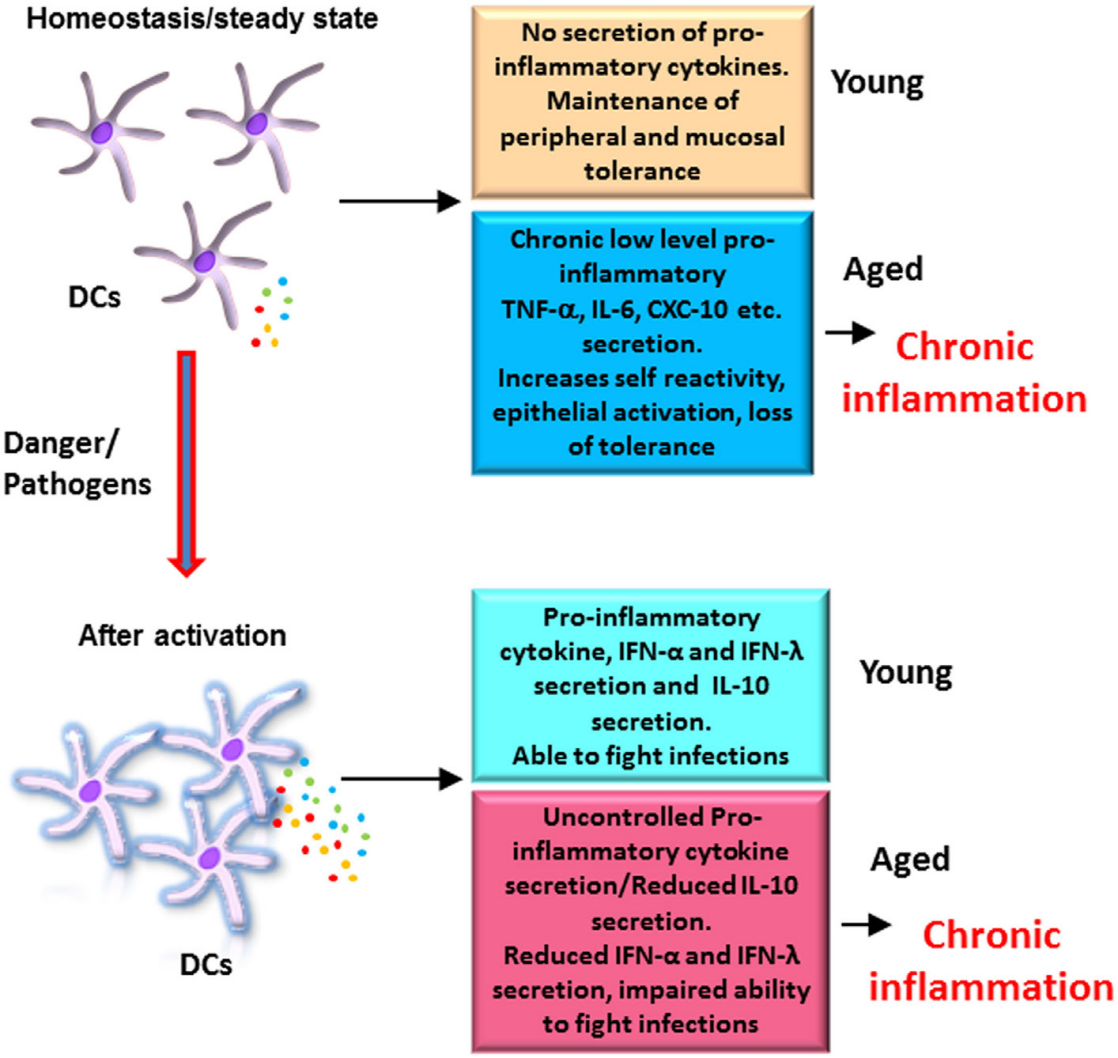
Altered functions of dendritic cells (DCs) from elderly contribute to chronic inflammation: DCs from elderly display an enhanced basal level of activation, which increases their reactivity to self-antigens, affects the function of epithelial barrier, and results in erosion of peripheral and mucosal tolerance at homeostasis. After activation with pathogens, DCs from elderly secrete enhanced levels of pro-inflammatory cytokines, which are not regulated as the secretion of anti-inflammatory cytokine IL-10 is impaired. This also contributes to inflammation. In addition, secretion of protective cytokines such as the IFN-α and IFN-λ is also decreased resulting in a decrease in the ability of elderly to fight infections. Figure depicts the differences in the response of DCs from aged and young subjects at homeostasis and after activation.

In contrast to mDCs in circulation, we have observed that MoDCs display increased secretion of pro-inflammatory cytokines TNF-α, CXCL-10, and IL-6 in response to TLR4 ligand, LPS (21). We also observed similar increase in these mediators in response to *Chlamydophila pneumoniae* (34). This increase was attributed to decrease in signaling *via* the PI3kinase/Akt pathway, which functions as a negative regulator of TLR signaling. Increased PTEN expression in DCs from aged subjects was found to be responsible for the deficient function of AKT (21). Most remarkably, we also observed a significant age-associated decrease in the secretion of the anti-inflammatory cytokine, IL-10, which is required to regulate inflammation. This defect in IL-10 secretion by DCs from aged subjects was a consequence of an inherent defect in DCs as addition of IL-10-inducing agents such as lithium chloride was unable to restore the production of IL-10 in DCs from aged subjects (35). This decrease in IL-10 production may also contribute to loss of tolerance in aging. In addition to impaired IL-10 production, DCs from aged subjects also displayed a defect in the production of type I and III interferons in response to both influenza and *Chlamydophila* (34, 36). The decrease in type I IFN production in older donors was also observed in MoDCs infected with West Nile virus (32). Interferons, both type I and III, are essential for blocking viral replication and preventing spread of infection (37, 38). In addition, the interferons also act on various cells to induce an antiviral state. Though the actions of both interferons are similar, type III interferons play a more important role in protection against infections of the mucosal surfaces. This is because the receptors for type I interferons are present on nearly all cells of the body, while type III display a more restricted pattern of expression with the receptors being present mainly on mucosal tissues (38). Type III IFNs thus contribute to the control of viral infections in epithelial cells of both respiratory and gastrointestinal tracts. Together, MoDCs from aged subjects display a selective deficiency in anti-inflammatory and protective cytokines accompanied with an increase in pro-inflammatory cytokines and chemokines (Figure 1).

## DCs and Impaired Vaccine Responses in the Elderly

Vaccine responses in the elderly are also compromised (39, 40). Reduced responses to influenza vaccines are well documented (40). Elderly display deficiency in mounting efficient immune response to new antigens or vaccines, but their capacity to generate recall responses to previously primed antigens is relatively better preserved (41). The underlying mechanisms are not well understood; in particular, the possible contribution of age-associated DC dysfunction has not been examined in humans. Successful response to vaccines requires presentation of the antigens by DCs as well as costimulation (42). DCs are important not only for the initiation of T cell responses but also for the generation of efficient effector and memory responses (43–47). Reduced phagocytosis as well as impaired migration of DCs from aged subjects may be one of the factors for the decreased vaccine responses (21). Furthermore, DCs also need to upregulate class I and II MHC molecules as well as costimulatory molecules, CD40, CD80 as well as CD86 for efficient antigen presentation. The response of human monocyte-derived DCs to TLR ligands indicates that the capacity of DCs from elderly to upregulate MHC and costimulatory molecules is not impaired (21, 25, 26). This is in contrast to murine DCs where reduced expression of CD80, CD86, and MHC II has been observed in DCs before and after infection (48–50). The discrepancy could be due to difference in species. However, most of the information is derived from MoDCs, and the expression of costimulatory molecules on human DC subsets in circulation or in tissues after stimulation/infection has not been investigated. It is possible that these DCs may behave differently compared to MoDCs. MoDCs from young and aged subjects have also been reported to have similar stimulatory capacity to induce proliferation of T cell lines developed in long-term cultures (26). However, during respiratory syncytial virus infection, decreased IFN-γ-producing cells were observed in response to MoDCs from old individuals (51). We have observed increased proliferation as well as IFN-γ secretion by T cells primed with MoDC from older subjects in the absence of stimulation supporting the enhanced activated state of DCs in the elderly (34). However, MoDCs from aged subjects were not as efficient as MoDCs from young subjects in enhancing the T cell proliferation and IFN-γ secretion after stimulation (34). In another study also, elderly adults displayed a reduced ability to prime antigen-specific CD8^+^ T cells (52). Furthermore, the expression of perforin and granzyme B was also reduced in the primed CD8^+^ T cells. Reduced T cell proliferation has also been reported in another study where TLR agonist-stimulated PBMCs (minus CD3 T cells) from older subjects’ induced lower proliferation of allogeneic adult T cells compared to stimulated PBMCs from adult subjects (22). In both these studies, reduced priming by DCs was believed to be one of the factors for the observed decreased activation of T cells, though direct priming of T cells by DC was not performed. In addition to MoDCs, we also observed a reduction in the capacity of pDCs from elderly to prime CD4 and CD8 T cell responses after stimulation with influenza (31). Altogether, these studies suggest that DCs from elderly display a deficiency in priming naïve T cell responses after stimulation. Preexisting inflammation in aged subjects could be one of the possible mechanisms, which modifies DC responses and reduces their capacity to prime T cells. This is supported by a recent clinical trial using an mTOR inhibitor, RAD001, which reduces inflammation. Treatment of older subjects with RAD001 prior to influenza vaccination was demonstrated to decrease the percentage of PD-1-positive CD4 and CD8 T cells compared to placebo and enhance the response to influenza vaccination (53). Enhanced basal level activation was also demonstrated to be responsible for the reduced response to yellow fever vaccine of African subjects compared to European subjects (54). Strategies to reduce inflammation prior to vaccination may therefore prove useful in enhancing vaccine responses in the elderly.

Emerging evidence indicates that different subsets of DCs may display differential ability to prime CD4 and CD8 T cell responses. For example, CD1c DC subset is believed to express high levels of molecules such as Ifi30 (GILT), HLA-DMA, and cathepsin H, which renders them more efficient in priming CD4 T lymphocyte responses (55). On the other hand, CD141^+^ mDC subset possesses a superior capacity to cross-present antigens to CD8 T lymphocytes (56, 57). We have observed reduced percentages of CD141^+^ mDC subset in the circulation of the elderly, but the alterations in DC subsets in tissues have not been studied (23). In this regard, a recent study by Yu et al. (58) has used a human CD34^+^ hematopoietic progenitor cells reconstituted immunodeficient mice model to examine the CD8^+^ T cell priming capacity of human respiratory CD1c^+^ and CD141^+^ DCs against intranasal live-attenuated influenza virus vaccination. Their results indicate that both DC subsets were efficient at activating antiviral CD8^+^ T cell responses against influenza. Nevertheless, lung CD1c^+^ DCs induced the expression of CD103 on the CD8 T cells, which allowed their retention in the lung epithelium and generate tissue-resident memory cells. Antigen-presenting capacity of different DC subsets in the elderly thus needs to be examined not only because of their capacity to prime different T lymphocyte subset but also since emerging evidence indicates different vaccines activate different DC subsets, and this differential activation is required for efficient adaptive immune responses (59). Fluzone was demonstrated to primarily activate MoDCs, while pneumovax activated monocytes. In contrast, Gardasil induced the activation of CD1c^+^ blood DCs (59). The specialization of APCs in response to different vaccines will have to be kept in mind when designing vaccines for the elderly. The response of different DC subsets to vaccine antigens and adjuvants may be determined to obtain information about the induction of adaptive immunity. For example, to improve vaccine response to fluzone in the elderly, it may be beneficial to design adjuvants that activate monocytes. In addition to the above parameters, the strength and duration of antigen priming by DCs also affects memory generation. A recent murine study with vaccinia virus demonstrated that cross presentation of antigens by DCs to TCR-identical cells leads to generation of tissue-resident memory CD8^+^ T cells versus the circulating memory cells (60). Studies examining the generation of tissue-resident memory in human subjects are not feasible due to ethical considerations. However, use of reconstituted humanized mice as described above (58) may be one of the approaches to examine tissue-specific human responses. Another approach is the emerging field of systems vaccinology (61), which may provide insight into the transcriptional and epigenetic signatures that could be predictive of reduced vaccine responsiveness in the elderly and may help design strategies to overcome the deficiencies.

## DCs and Damage-Associated Molecular Patterns (DAMPs)

In contrast to exogenous danger threats, there is not much known about the response of DCs from aged subjects to endogenous, DAMPs. DAMPs are released upon cell or tissue damage, and enhanced tissue damage is a characteristic of aging. The inflammasome pathway plays a major role in recognizing a wide array of DAMPS including cholesterol crystals, uric acid crystals, extracellular ATP, amyloid-beta, and ceramides and lipids (62, 63), many of these molecules accumulate during aging (64). Studies in mice have linked impaired glucose tolerance and cognitive decline to enhanced expression of NLRP3 inflammasome pathway during aging (65). The increased TNF-α levels in aged mice were reported to enhance the expression of NLRP3 inflammasome in adipose tissue and liver, which results in impaired glucose tolerance. A recent study (66) in humans has demonstrated a direct correlation between specific inflammasome expression modules and age-related diseases such as hypertension, as well as with diminished longevity. The study identified two metabolites, adenine and *N*^4^-acetylcytidine, which prime and/or activate the NLRC4 inflammasome and induce hypertension and inflammatory signatures. These studies indicate an enhanced activity of the inflammasome pathway during aging; however, these changes have not been determined in DCs. Aging also leads to changes/loss in protein homeostasis, proteostasis due to intracellular damage, which contributes to the pathogenesis of neurodegenerative diseases such as Alzheimer’s disease (67). It would be interesting to determine the response of DCs from healthy aged subjects to some of the pathological proteins such as amyloid-beta and compare it to response of DCs from AD patients.

## DCs and Tolerance

Maintenance of tolerance against self-antigens is another primary function of DCs (68). DCs are constantly being exposed to self-antigens generated during cell death, tissue injury, etc. Under steady-state conditions, DCs in the periphery uptake these antigens but do not get activated. Presentation of self-antigens to T cells in the absence of costimulatory molecules or activation signals leads to T cell tolerance *via* T cell anergy or induction of T regulatory cells. However, if the environment is inflamed and the DCs are activated, they may present self-antigens to T cells to generate an immune response against self-antigens. This can lead to autoimmunity and inflammation. Since our group and others (18, 69) have observed that DCs from aged subjects secrete low basal levels of pro-inflammatory cytokines, it is suggested that the DCs are activated. Indeed, we observed increased basal level of NF-κB activation in DCs from aged subjects (70). Furthermore, we also found increased reactivity of DCs from aged subjects to self-DNA. Self-DNA is released when apoptotic cells’ clearance is defective and cells undergo secondary necrosis. In keeping with this, DCs from aged subjects exhibited reduced uptake of apoptotic cells (70). The increased immune response instead of tolerance to self-antigens contributes to the inflammation during aging (Figure 1).

In addition to peripheral tolerance, DCs also play a role in maintaining tolerance at mucosal surfaces (71). The lung and gut mucosa are constantly exposed to harmless and innocuous antigens in the form of particles from inhaled air and food. Furthermore, the commensal microbial communities present in the oral and gastrointestinal mucosa are essential for human health and thus an immune response against them would be detrimental. Significant progress has been made in the last few years identifying DCs as critical mediators of tolerance induction at these surfaces. The epithelial cells in the mucosa secrete factors such as retinoic acid and TGF-β, which act upon DCs to induce tolerance to prevent response against harmless antigens and commensal microbiome (71–73). Our studies suggest that DCs from aged subjects display impaired response to retinoic acid and are deficient in inducing T regulatory cells for tolerance (23). The infections of the respiratory mucosa such as the influenza and *Chlamydia pneumoniae* are more prevalent and severe in the elderly (74). The older population is also more susceptible to bronchitis, asthma, COPD, and emphysema (74–76). Reduced capacity of DCs from aged subjects to maintain tolerance in the airways may enhance inflammation and invasion by pathogens due to impaired remodeling of the airways. Furthermore, we have also demonstrated that the basal level of activation of DCs from aged subjects leads to low-level secretion of pro-inflammatory cytokines, which activates the epithelium even in the absence of infection (69). Exposure of airway epithelial cells to supernatants from unstimulated DCs from aged subjects, but not young subjects, led to an increase in permeability of the epithelial barrier, which was accompanied with secretion of chemokines and upregulation of activation molecules. Therefore, DCs from aged subjects are not only defective in their response to tolerogenic signals from epithelial cells but also act on the epithelium to compromise its barrier functions.

Although studies were performed with airway epithelial cells and DCs, a similar process may be occurring at the level of gut and skin. Infections of both these surfaces are also more common in the elderly (77). For example, *Helicobacter pylori* and *Clostridium difficile* infections of the gut are often more severe and result in hospitalization of the elderly (78). It is also well established that with advancing age there are significant changes in the composition of gut microbiota with increase in Gram-negative bacteria, like *Enterobacteriaceae* and other pathogens (79). These Gram-negative bacteria secrete lipopolysaccharides, and we have previously reported that inflammatory response to LPS is enhanced with age (21). In addition, the level of short-chain fatty acids (SCFAs), such as acetate, butyrate, and propionate, are also reduced in the intestine of aged subjects as compared to young subjects (80). SCFAs synthesized by gut microbiota can act on DCs to prevent their activation and enhance their capacity to induce T regulatory cells to maintain tolerance in the gut (81). Enhanced inflammation in the gut increases the susceptibility of the elderly to gastrointestinal infections. Similar to gut infections of the skin including viral infections like herpes zoster (shingles), pressure ulcers, bacterial, or fungal infections, methicillin-resistant *Staphylococcus aureus* are prevalent in the elderly. Aberrant functions of DCs at the mucosal surfaces may account for the increased mucosal infections observed in the elderly.

## Potential Mechanisms Responsible for DC Dysfunction in the Elderly

The above studies highlight the age-associated alterations in DC functions; however, the mechanisms responsible for the changes are not well understood. Changes in signaling mechanisms such as enhanced basal level activation of NF-κB are thought to be responsible for the increased inflammatory responses of the DCs from the elderly. This has been observed both in MoDCs and circulatory DCs (18, 21, 70). Nevertheless, it is still not defined whether the changes are due to an intrinsic defect in DCs or external senescent microenvironment drives the changes. Evidence regarding both mechanisms is present. The age-associated increased circulatory pro-inflammatory mediators such as TNF-α, prostaglandins can cause DCs to mature and secrete pro-inflammatory cytokines at homeostasis. This process can start during DC differentiation in the bone marrow since the fat content in bone marrow has been shown to increase with age (82), and adipocytes are major producers of pro-inflammatory cytokines (83). DC intrinsic mechanisms such as epigenetic changes including chromatin and methylation alterations or changes in micro RNA may also account for observed changes in DC function. We have observed increased binding of type I and type III IFN promoters to inhibitory histone, H3K9 (36), but studies comparing the chromatin accessible elements at a global level have not been performed in DCs from aged and young subjects. The inability to obtain sufficient number of DCs from the blood of the elderly has been a major hindrance. The advent of novel next generation techniques such as ATAC-seq (84) as well as single-cell sequencing (85) may enhance the feasibility of performing such studies. Similarly, changes in DNA methylation in T lymphocytes have been well documented in aging (86). However, methylation changes in DCs have not been studied in aged subjects. This is an area of importance for future studies as methylation has a significant impact on gene function (87). Studies focused on age-associated alterations in the expression of long non-coding RNAs (88) are also of potential importance to understand the mechanisms underlying DC dysfunction in the elderly.

## Conclusion

In summary, aging impacts DC functions in multiple ways. The enhanced activated state of DCs from elderly leads to the erosion of peripheral and mucosal tolerance and induction of inflammation. The secretion of inflammatory cytokines by activated DCs is also uncontrolled due to a defect in IL-10 secretion. In addition, the secretion of type I and III interferons is compromised, which enhances the susceptibility of the elderly to viral and bacterial infections. More studies are required to understand the effect of age on various DC subsets particularly in the tissues.

## Author Contributions

AA wrote the review with the help of SA and SG.

## Conflict of Interest Statement

The authors declare that the research was conducted in the absence of any commercial or financial relationships that could be construed as a potential conflict of interest.
